# Retrospective Analysis of Emergency Medical Services (EMS) Physician Medical Control Calls

**DOI:** 10.5811/westjem.2020.1.44943

**Published:** 2020-04-22

**Authors:** Balaj Rai, Joseph Tennyson, R. Trevor Marshall

**Affiliations:** * The Christ Hospital, Department of Internal Medicine, Cincinnati, Ohio; † University of Massachusetts Medical School, Department of Emergency Medicine, Worcester, Massachusetts; ‡ Stony Brook University, Department of Emergency Medicine, Stony Brook, New York

## Abstract

**Introduction:**

Although emergency medical services (EMS) standing-order protocols provide more efficient and accurate on-scene management by paramedics, online medical direction (OLMD) has not been eliminated from practice. In this modern era of OLMD, no studies exist to describe the prevalence of reasons for contacting OLMD.

**Objectives:**

The primary goal of this study was to describe the quantity of and reasons for calls for medical direction. We also sought to determine time diverted from emergency physicians due to OLMD. Finally, we hoped to identify any areas for potential improvement or additional training opportunities for EMS providers.

**Methods:**

This was a descriptive study with retrospective data analysis of recorded OLMD calls from January 1, 2016, to December 31, 2016. Data were extracted by research personnel listening to audio recordings and were entered into a database for descriptive analysis. We abstracted the date and length of call, patient demographic information (age and gender), category of call (trauma, medical, cardiac, or obstetrics), reason for call, and origin of call (prehospital, interhospital, nursing home, or discharge).

**Results:**

The total number of recordings analyzed was 519. Calls were divided into four categories pertaining to their nature: 353 (68.5%) medical; 70 (13.6%) trauma; 83 (16.1%) cardiac; and 9 (8%) were obstetrics related. Repeat calls regarding the same patient encounter comprised 48 (9.4%) of the calls. Patient refusal of transport was the most common reason for a call medical direction (32.3% of calls). The total time for medical direction calls for the year was 26.6 hours. The maximum number of calls in a single day was seven, with a mean of 2.04 calls per day (standard deviation [SD] ± 1.18). The mean call length was 3.06 minutes (SD ± 2.51).

**Conclusion:**

Our analysis shows that the use of OLMD frequently involves complex decision-making such as determination of the medical decision-making capacity of patients to refuse treatment and transport, and evaluation of the appropriate level of care for interfacility transfers. Further investigation into the effect of EMS physician-driven medical direction on both the quality and time required for OLMD could allow for better identification of areas of potential improvement and training.

## INTRODUCTION

Emergency medical services (EMS) systems often require online medical direction (OLMD) by trained physicians to supplement protocol-based management of patients in the field.^1^,^2^ Over the past few decades the EMS system has evolved to employ standing-order protocols through offline medical direction, providing more efficient and accurate on-scene management by paramedics without the need for OLMD.^3^ Protocol-based care has been shown to be at least equivalent to OLMD-based care in urban EMS systems.^4^ On average a call to OLMD takes approximately four minutes of physician time per call and results in increased on-scene time and delay in arrival to the hospital.^5^,^6^

OLMD is designed to provide EMS personnel with access to a physician to address more complicated clinical issues such as interfacility transfer authorization and guidance of care, ventilator and advanced medication management, patient refusals, and evaluation of a patient’s medical decision-making capacity. These calls are often related to OLMD contact for refusal of medical aid due to provider concerns about a patient’s medical decision-making capacity or in the case of high-risk diagnoses. Previous studies have demonstrated a benefit to physician involvement.^7^–^9^

A few studies have explored the efficacy of using an OLMD vs protocol-based hospital care. Erder et al determined that paramedic discretion to correctly triage OLMD use would result in shorter on-scene times for most patients. In one study, 86% of patient-care interventions were provided based on standing orders with an overall paramedic error rate of 0.6%.

The primary goal of this study was to determine the most common causes for contact of medical control. Additionally, we aimed to determine the time diverted from emergency physicians due to OLMD. In reviewing these data we hoped to identify possible areas for improvement and additional training opportunities for EMS respondents. We hypothesized that given the success of standing-orders protocols, the remaining needs for medical control would be more complex and require longer online times.

## METHODS

State regulations require that all OLMD be performed by licensed, credentialed physicians and be recorded. In our system, EMS providers can contact OLMD through one of two means. Providers may contact the destination emergency department via a central two-way radio-based system referred to as central medical emergency direction (CMED) or providers affiliated with our medical center may contact an EMS physician via a dedicated voice over Internet protocol (VOIP) toll-free telephone number when seeking medical control. Calls through the VOIP system are routed to the EMS physician on call using an Internet-based call forwarding software (RingCentral, Belmont, CA). All recordings are stored on a secure, password-protected server.

Institutional review board approval was obtained to review 12 months of this existing VOIP call data. Researchers reviewed all recorded calls within the study period of January 1, 2016, to December 31, 2016. Data were extracted by research personnel listening to audio recordings and were entered into a secure database for descriptive analysis. The research protocol called for exclusion of any call whose audio quality was insufficient for extraction. No other exclusion criteria were included in the protocol.^10^,^11^

Our group provides medical oversight for services, responding to over 120,000 calls per year. The OLMD system is covered 24 hours per day by either an EMS fellow with available faculty backup or by EMS faculty. OLMD is provided to a variety of different prehospital and interfacility services including private services providing both emergency response and interfacility transfer, helicopter emergency medical services (HEMS), and both hospital-owned-and-operated and fire-based services providing emergency response.

**Population Health Research Capsule**

What do we already know about this issue?

Prior studies have shown improved scene times and equal quality with reduction of online medical direction (OLMD) in favor of standing order protocols. Refusal of care is a common reason for an OLMD call.

What was the research question?

In a large diverse multi-department medical direction system, what are the reasons for calls to OLMD?

What was the major finding of the study?

The most common reason for calls to OLMD was refusal of medical aid. OLMD was able to effect a change in the patient’s decision in 10% of the cases they spoke to.

How does this improve population health?

This study helps to build knowledge about the use of OLMD by EMS services, so system designers can plan appropriate staffing and infrastructure.

Many of the calls in this system are for OLMD for interfacility transport. Massachusetts Statewide Treatment Protocols state:

“In cases where the patient’s care during the transfer exceeds the standing-order scope of practice as defined by the current version of the Statewide Treatment Protocols for an EMT-Paramedic or the patient is unstable or is likely to become unstable as defined previously (see “Scope of Practice” above) will provide a concise, complete and accurate patient report to an On-Line Medical Control physician…”^12^

As a result, many calls are placed to OLMD for interfacility transfers. While EMS providers are permitted to obtain refusals of medical aid independently, they are required to call in cases of refusal in the context of established invasive care (ie, refusal following dextrose administration in hypoglycemia). Crews are also encouraged to call for cases where there is a concern for the medical decision-making capacity of the refusing party or in cases considered to be high risk for patient morbidity.

We collected and managed study data using REDCap electronic data capture tools hosted at the University of Massachusetts Medical School.^13^ We abstracted the date, time and length of call, patient demographic information (age and gender), category of call (trauma, medical, cardiac, or obstetrics), reason for call, and origin of call (prehospital, interhospital, nursing home, or discharge). Call categories were defined prior to review based on expected call categories and experience in provision of OLMD in this area. Consideration was given to the difference in nature of prehospital and interfacility calls of a general medical as opposed to cardiac nature, as well as the separation of these categories in the statewide treatment protocols. Because of this, cardiac calls, although arguably a subset of medical calls, were given their own category. The category of “reason for call” was meant to determine the specific support requested of the OLMD from the field providers. Level of care in this context refers to OLMD assistance in determining the appropriate level of the transporting EMS service; Basic Life Support, Advanced Life Support, or critical care.

Paramedic-level ambulances performing interfacility transfers are required to have transport ventilators and may continue established ventilator settings. These calls also require OLMD. Ventilator sedation referred to requests for orders to manage the sedation of patients being transported on the ventilator. Ventilator management requests involved the request for orders regarding settings for the ventilator. Termination of resuscitation calls were for the order to terminate resuscitative efforts for cardiac arrests in the field. This order from OLMD is required by state protocol when the termination protocol is employed. In contrast to this, confirmation of death/do no resuscitate (DNR) was a category applied to calls where OLMD was notified in cases where a valid DNR order was in place. This is not required by state protocol. The other category also lists several calls from the HEMS service to provide policy-based approval to perform missions by ground when the aircraft could otherwise be available (often due to local weather conditions). This is a policy of our specific service and was a source of enough calls to warrant breaking them out into a separate category. The categories of “reason for call” were determined a priori, but the subcategories of “other” were determined during the review process.

We also abstracted the time of day calls were placed and their durations. The database was created by the principal investigator. We conducted analyses using JMP Pro 13 (SAS Institute Inc., Cary, NC).

## RESULTS

Based on estimates of annual volume of the EMS services included in our analysis, OLMD was sought in less than one percent of responses. The total number of recordings available in the study period was 519. No calls were excluded. Calls were received from 17 distinct services. Private services placed the most calls at 60%; HEMS 18%, hospital-based 13%; and fire-based 9%. The four services calling the most frequently composed 84.4% of the calls.

The distribution of age and gender of the patients is shown in Table 1. Calls were divided into four categories pertaining to their nature: 353 (68.5%) medical; 70 (13.6%) trauma; 83 (16.1%) cardiac; and 9 (8%) obstetrics. Repeat calls regarding the same patient encounter comprised 48 (9.4%) of the calls.

**Figure 1:**
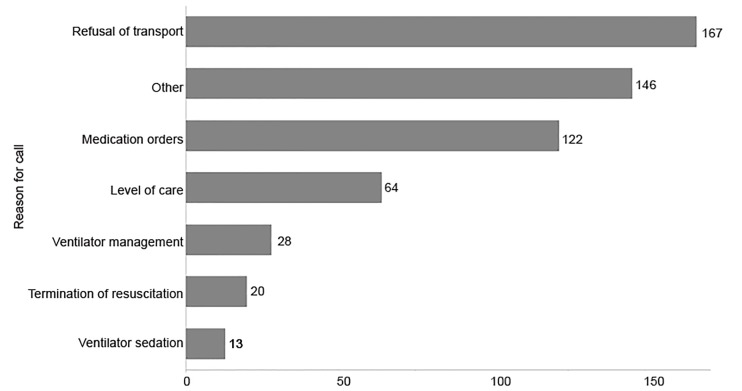
Reasons for calls for medical direction.

The reasons for the call for medical direction are displayed in Figure 1. Refusal of medical aid was the most common reason for calling OLMD with 167 (32.3%) of the 519 reviewed calls categorized in this manner. Of the 167 calls, the OLMD physician spoke directly to the patient in 48 (30%) of cases and effected a change in the patient’s decision to refuse in 5 (10.5%) cases. Some of these calls involved OLMD assessment of the patient’s medical decision-making capacity, though a quantitative analysis of this is not reported here. Reasons listed in the “other” category included administrative approval for the HEMS crew to perform ground critical care transportation, confirmation of death/DNR, and a change of destination as well as other, less frequent, reasons.

**Figure 2:**
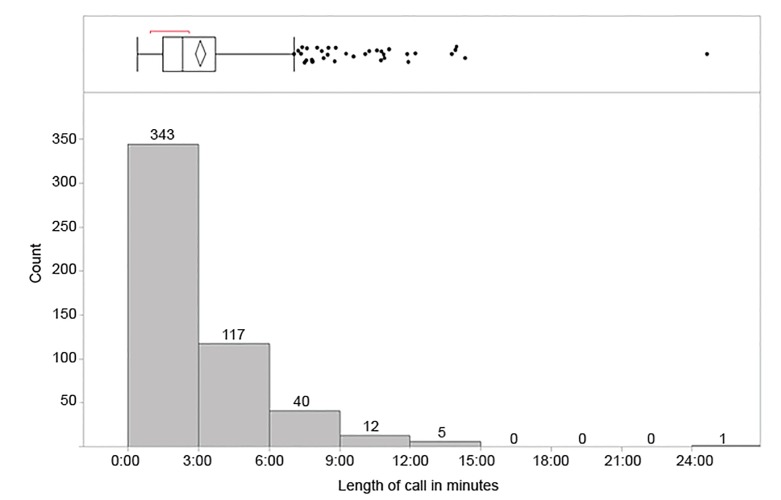
Distribution of call duration in minutes when prehospital providers call for medical direction. Figure is displayed as histogram of call duration with accompanying box plot (above) of median and interquartile ranges of the distribution, with dots representing outliers and diamond denoting the mean value.

**Figure 3:**
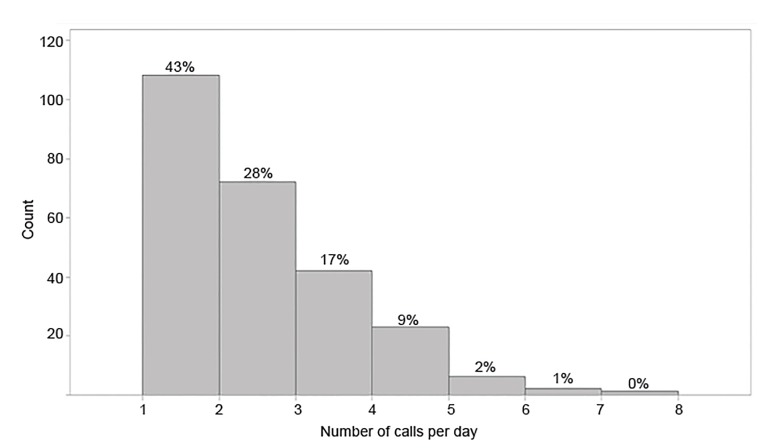
Distribution of number of calls per day placed by prehospital providers to base physician. Figure is displayed as a histogram of the number of calls to the medical direction service per day over the reviewed 12 months.

The total time for medical direction calls for the year was 26.6 hours. The maximum number of calls in a single day was seven, with a mean of 2.04 calls per day (standard deviation [SD] ± 1.18) (Figure 2). As may be seen from the box plot in Figure 2, the mean and median numbers of calls per day were very similar. The mean call length was 3.06 minutes (SD ± 2.51) (Figure 3). Calls to OLMD were placed between 1 AM–8 AM in 20.7% (107) of cases, between 8:01 AM–4 PM in 37.6% (194) of cases and between 4:01 PM–12 AM in the remaining 41.7% (215) of cases.

## DISCUSSION

This descriptive analysis of one year of medical direction calls identifies that a significant need for OLMD continues in our age of protocolized standing orders. Past research has shown that standing orders as opposed to OLMD can speed care without weakening quality.^11^ Our analysis shows that the use of OLMD frequently involves complex decision-making such as evaluation of the medical decision-making capacity of patients to refuse against medical advice, and evaluation of the appropriate level of care for interfacility transfers.

Our analysis further demonstrates the amount of time these calls to medical direction utilize. Our mean and median times are marginally shorter than previously reported data.^5^ EMS physician-based medical control provides an opportunity for a significant time savings to emergency physicians who would otherwise be required to provide this service – in the case of our data, a total of 1594 minutes over the course of a year. Additionally, the shorter average time for call completion compared to previously reported data suggests that routing calls to EMS-specialist physicians might provide an increase in overall efficiency in the medical direction process.

The most common reason for the call to OLMD was a patient refusal of medical aid. Prior published data indicates that speaking to an OLMD physician can improve the rate of transport of these against-medical-advice patients.^8^,^9^ Some of these calls involved assessment of the patient’s medical decision-making capacity. There is a paucity of existing research on the ability of prehospital providers to perform this assessment.^9^,^14^,^15^ A further evaluation of OLMD to determine the extent to which these calls involve capacity assessment may aid in future research as well as prehospital curriculum development.

In addition to prehospital decision-making, a large portion of calls pertained to interfacility transfers. Determination of appropriate level of care for interhospital transfers was 12.4% of calls, often complicated decisions requiring knowledge of system capabilities, prehospital protocols, and scope of practice beyond the knowledge base of the average emergency physician. Further, 7.9% of calls concerned ventilator management or sedation of patients prior to or during transfer. These calls often involve multiple aspects of out-of-hospital care, which can represent a significant burden for the emergency physician, especially as they may not be receiving this patient.

Previous studies have addressed the role of OLMD from the standpoint of prehospital providers, but no studies were identified examining the effect on the emergency physician of providing OLMD. The implementation of an EMS physician medical direction system may allow the diversion of some high-risk and high-complexity medical direction calls such as refusal of medical aid and other calls involving complex medical decision-making from EDs. This has the potential to decrease distractions and interruptions to EPs during clinical shifts.

## LIMITATIONS

Limitations of the study most prominently include a potential analytical bias due to subjective categorization of medical record calls based on the interpretations of one researcher. Furthermore, this study was conducted at a single, academic medical center with an academic EMS physician group, resources that are not available at many institutions.

## CONCLUSION

Most calls for OLMD involve complex decision-making such as refusal of medical aid and level of care determination for interfacility transfers. The implementation of an EMS-physician based OLMD model provided for the opportunity to decrease the time diverted from emergency physicians in order to provide OLMD to out-of-hospital providers as well as reducing the overall time required to provide OLMD. Further investigation into the effect of EMS physician-driven OLMD on both the quality and time required for OLMD could allow for better identification of areas of potential improvement and training.

## Figures and Tables

**Table 1:** Patient characteristics in study to determine the most common reasons prehospital providers call for medical direction.

Variables	Frequency (n = 516)	Mean (SD)
Age in years		55.3 (24.9)
Gender		Proportion
Male	229	46.5%
Female	248	50.3%
Unknown	16	3.2%

*SD*, standard deviation.
